# Supplementary material for: Alpha 7 nicotinic acetylcholine receptors signaling boosts cell-cell interactions in macrophages effecting anti-inflammatory and organ protection
Source: Commun Biol. 2023 Jun 23;6:666. doi: 10.1038/s42003-023-05051-2 (PMC10290099; doi:10.1038/s42003-023-05051-2)
Supplement: Supplementary file 2 — Supplementary Information [file 42003_2023_5051_MOESM2_ESM.pdf]

Supplementary Information

Supplementary Figures

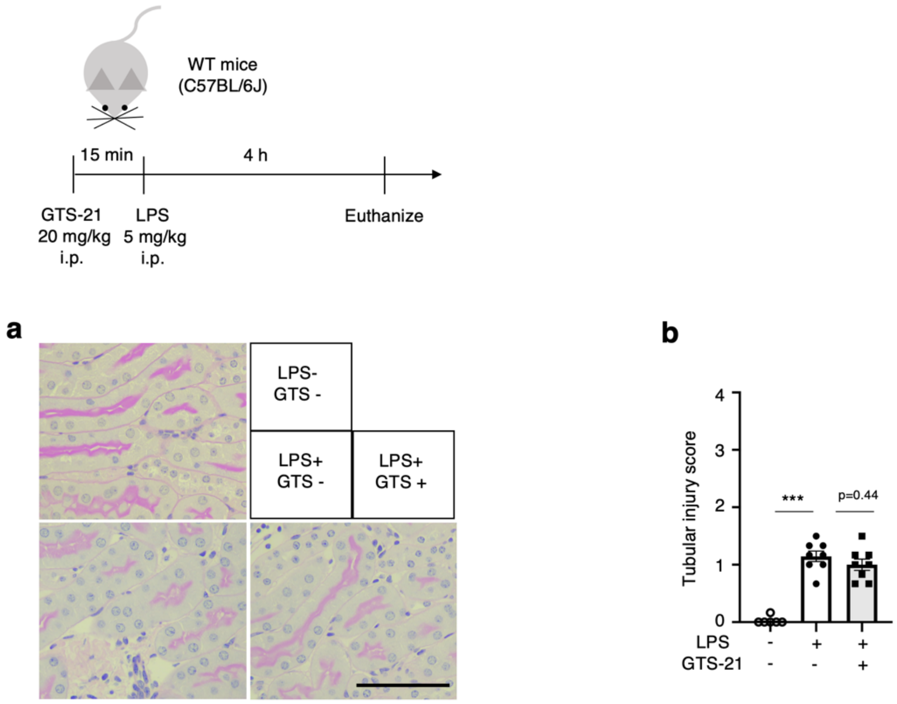

Supplementary Figure 1. Histological damages in WT mice kidney

(a) Representative pictures of PAS staining and (b) tubular injury score.

PAS staining revealed that LPS caused a slight increase in the flattening of the brush border (Fig.1a). No histological changes were observed following LPS administration with or without GTS-21 (Fig.1a and b).

Scale bar = 50 μm.

*P<0.05, **P<0.01, ***P<0.001, ****P<0.0001 (one-way ANOVA followed by Tukey's post hoc test). All data are presented as mean ± SEM. WT, wild-type; GTS-21, 3-(2,4-Dimethoxybenzylidene)- anabaseine dihydrochloride; LPS, lipopolysaccharide; PAS, periodic acid-Schiff; ANOVA, analysis of variance; SEM, standard error of the mean.

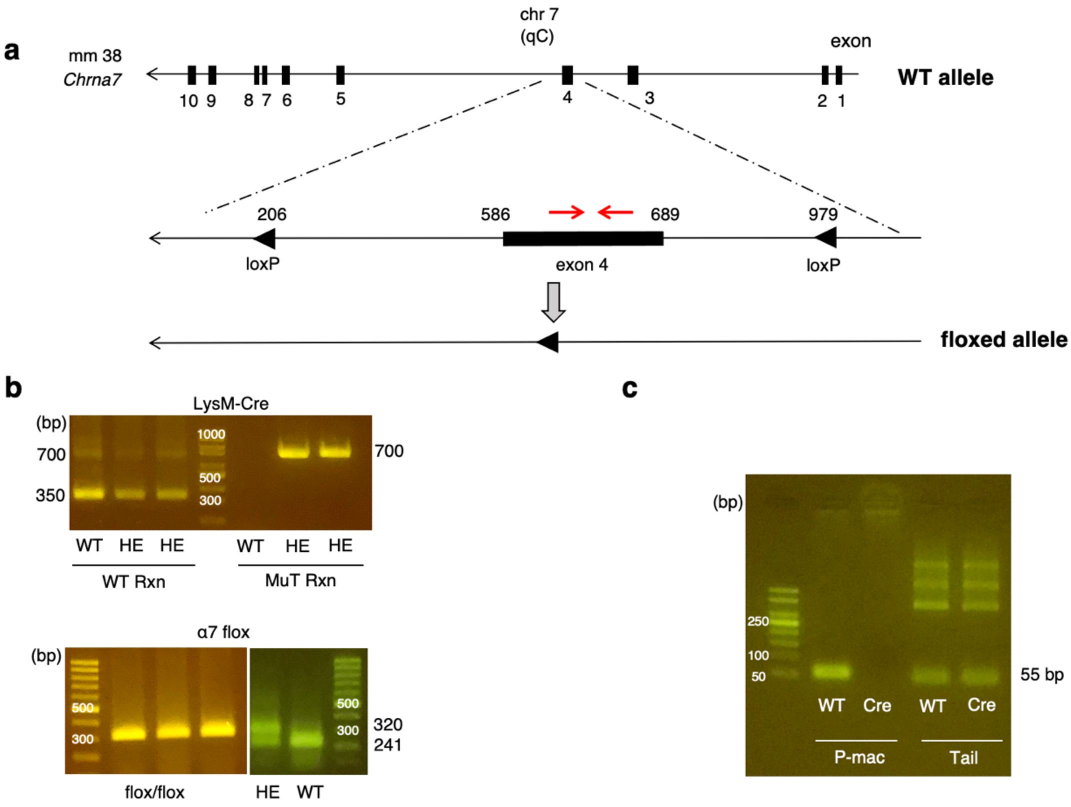

Supplementary Figure 2. Validation of the macrophage-specific α7nAChR KO mice

(a) The scheme of allele locus of *Chrna7*. Red arrows indicate primer sequences designed to detect exon4 that sandwiched between the LoxP site.

(b) Tail PCR differentiated WT and KO phenotypes. Tail PCR was performed following JAX genotyping protocols. LysM-Cre; WT = 350 bp, mutant = 700 bp, α7flox; WT = 241 bp, flox/flox = 320 bp.

(c) Confirmation of deletion of α7AChR (*Chrna7*) in macrophage. Peritoneal macrophages elicited by thioglycolate were collected and described in methods. DNA extracted from peritoneal macrophage and tail were amplified using a primer designed to detect exon 4 (red arrows in a).

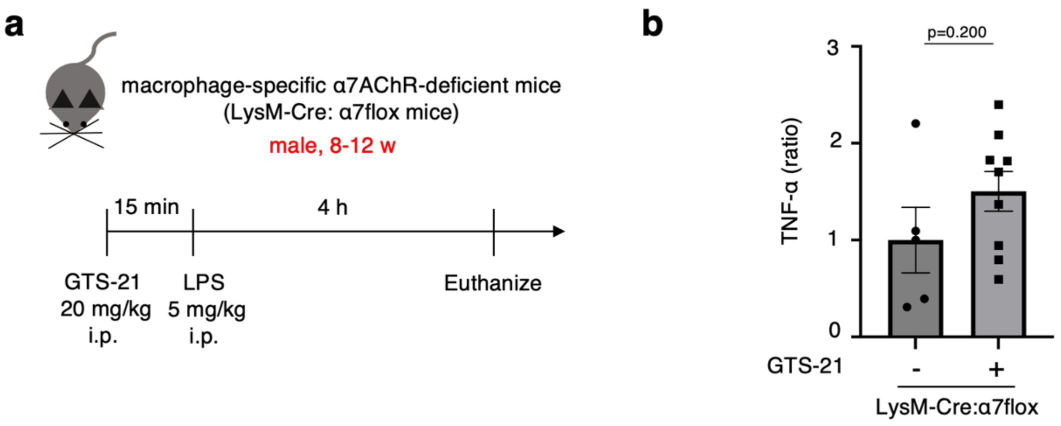

Supplementary Figure 3. Re-experiments of Figure 2

The experiment was retested with the exact sex and age of the mice.

(a) Experimental design. Only 8 to 12 weeks old, male LysMCre:α7flox mice were used in this re-experiments. N = 6-9, in each group.

(b) No significant difference in TNF-α (ratio) with GTS-21 administration (Unpaired T-test, p=0.2). GTS-21, 3-(2,4-Dimethoxybenzylidene)- anabaseine dihydrochloride; LPS, lipopolysaccharide; TNF-α, tumor necrosis factor α.

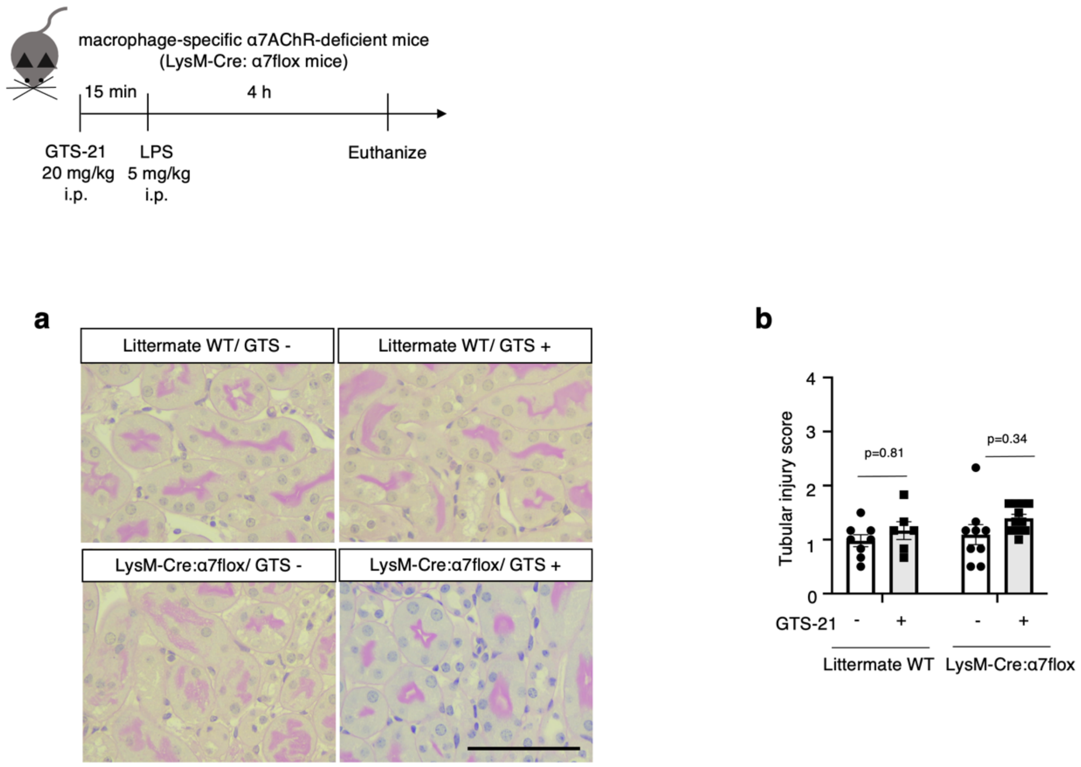

Supplementary Figure 4. Histological damages in LysMCre:α7flox mice

Representative images of PAS staining (a) and tubular injury scores (b).
Neither LysMCre: α7flox mice nor littermate WT mice showed significant differences in tubular injury scores after treatment with GTS-21.
Scale bar = 50 μm. *P<0.05, **P<0.01, ***P<0.001, ****P<0.0001 (two-way ANOVA followed by Tukey's post hoc test). All data are presented as mean ± SEM. WT, wild-type; GTS-21, 3-(2,4-Dimethoxybenzylidene)- anabaseine dihydrochloride; LPS, lipopolysaccharide; PAS, periodic acid-Schiff; ANOVA, analysis of variance; SEM, standard error of the mean.

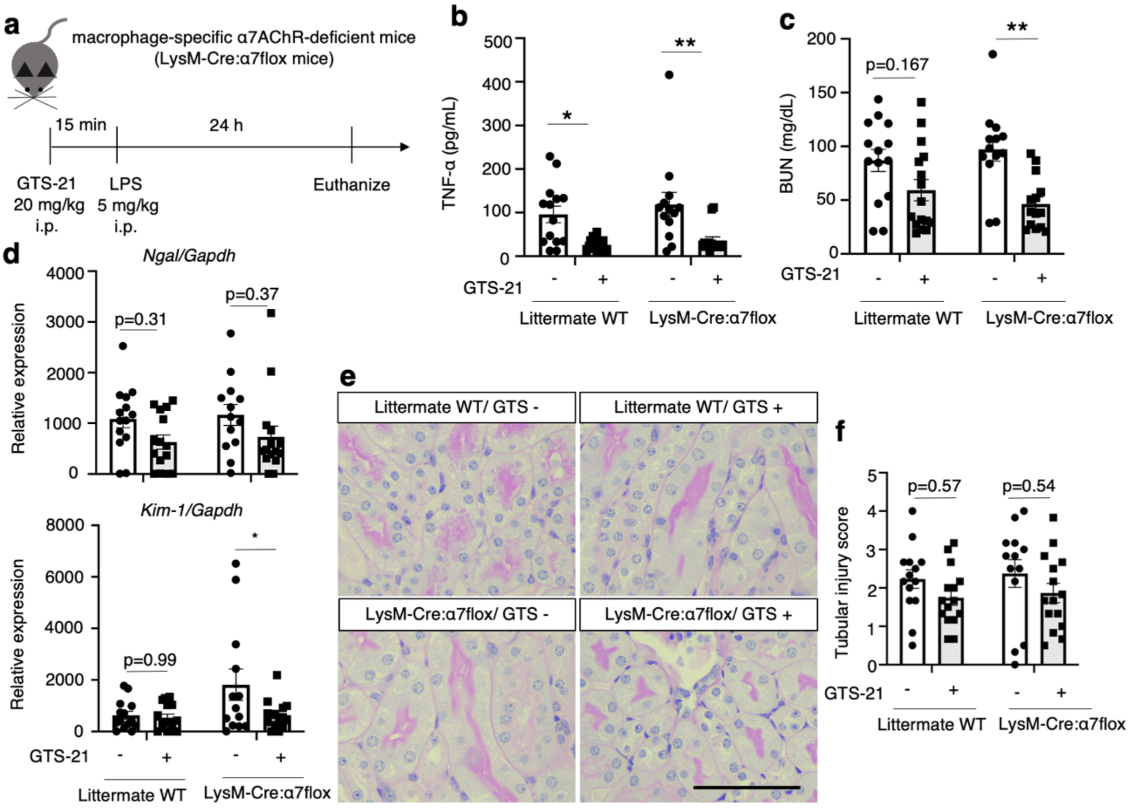

Supplementary Figure 5. GTS-21 has kidney protective effects even in macrophage-specific α7AChR-deficient mice in the late stage of injury.

(a)The study protocols. The mice received LPS administration after the GTS-21 injection, then 24 hours later they were euthanized, and blood and kidney samples were collected. (b) Both Littermate WT and macrophage-specific α7nAChR KO mice had decreased TNF-α levels after treatment with GTS-21 (n=13–16 in each group). (c–d) Plasma BUN (c), *Ngal* (d), and *Kim-1* (e) were not significantly suppressed by GTS-21 treatments in littermate WT mice, but BUN and *Kim-1* were decreased by GTS-21 treatments in macrophage-specific α7nAChR KO mice. (d) Protection against LPS-induced kidney injury by GTS-21 (n=6–11 in each group). (e, f) Representative images of PAS staining and tubular injury scores. Scale bar = 50 μm. *P<0.05, **P<0.01, (two-way ANOVA followed by Šidák's multiple comparisons tests (b) and Tukey's post hoc test (c, d, and f)). All data are presented as mean ± SEM. WT, wild-type; GTS-21, 3-(2,4-Dimethoxybenzylidene)- anabaseine dihydrochloride; LPS, lipopolysaccharide; TNF-α, tumor necrosis factor α; BUN, blood urea nitrogen, PAS, periodic acid-Schiff; ANOVA, analysis of variance; SEM, standard error of the mean.

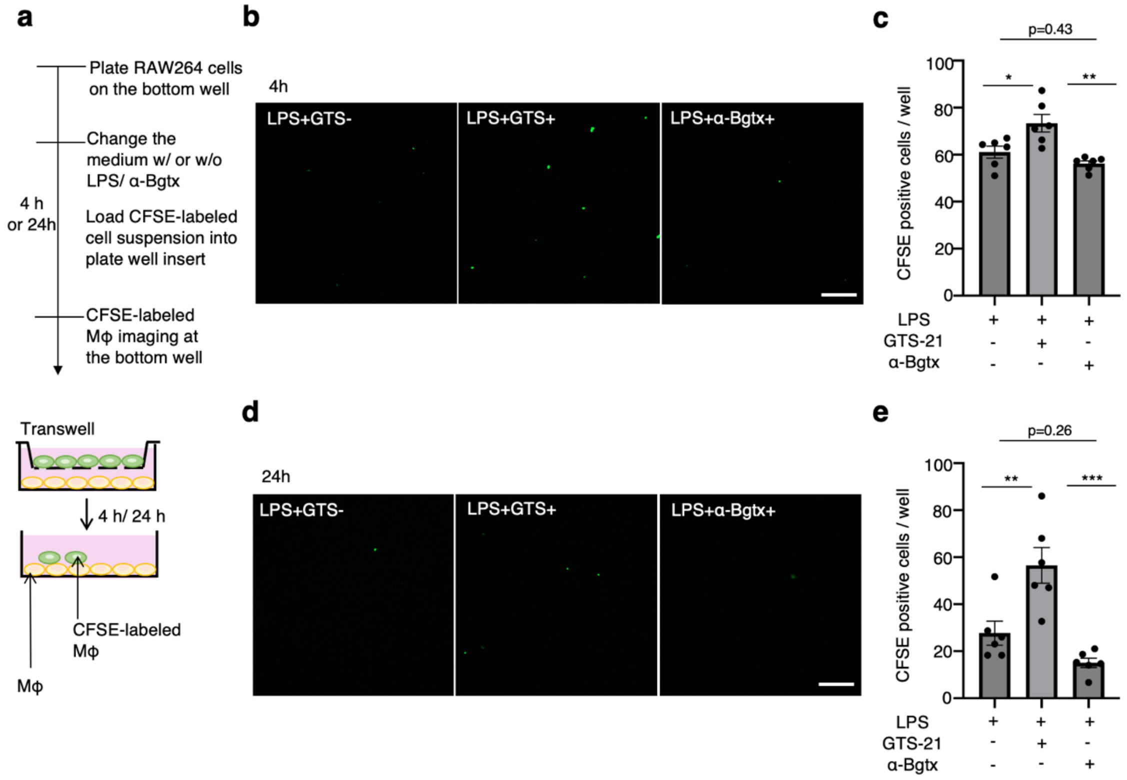

Supplementary Figure 6. Alpha-Bgtx reduced macrophage contacts increased by GTS-21 in transwell migration assay.

(a) The schema of this experiment. Migration assay of CFSE-labeled RAW 264 cells using a transwell system in different conditions. After 4 hours or 24 hours of incubation with and without LPS and α-Bgtx (1μg mL^{-1}), the bottom wells were photographed. (b-e) Representative images of migrated cells in (b; 4h) and (d; 24h). Bar graphs show the quantitative data of CFSE-positive migrated cells (c; 4h. e; 24h).

Scale bar= 150 μm.

*P<0.05, **P<0.01, ***P<0.001, (one-way ANOVA followed by Tukey's post hoc test). All data is presented as mean ± SEM. LPS, lipopolysaccharide; α-Bgtx, α-bungarotoxin; CFSE, Carboxyfluorescein succinimidyl ester; ANOVA, analysis of variance; SEM, standard error of the mean.

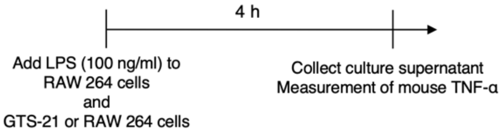

	(a)	(b)	(c)	(d)
Added cells or reagents	-	GTS-21	RAW264	-
# of RAW 264 (x10^4)	6	6	6	12

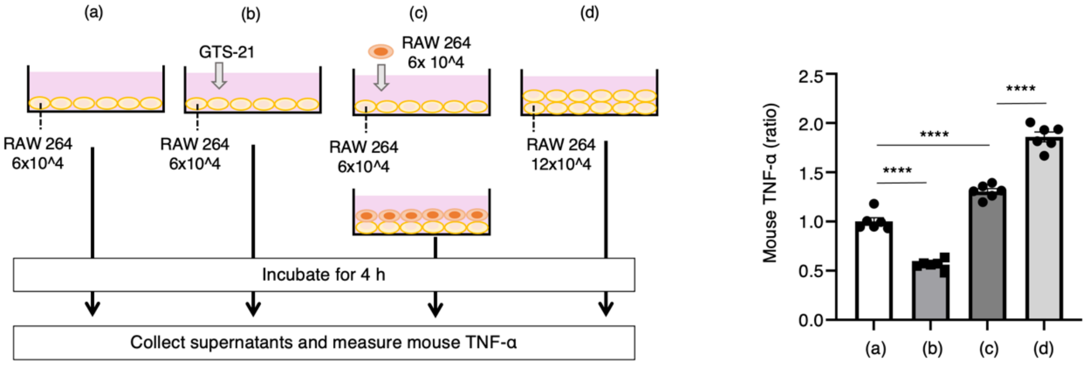

Supplementary Figure 7. Co-culture of RAW 264 cells suppressed LPS-induced TNF-α productions.

TNF-α produced by RAW 264, murine macrophage cell, was examined under the following conditions.

(a) 6 x 10^4 per well cells (for control)

(b) 6 x 10^4 per well cells with GTS-21 (100 μM)

(c) 6 x 10^4 per well cells with additional the same number of RAW 264 cells

(d) 12 x 10^4 per well cells

Comparing (a) and (d), the level of TNF-α produced approximately doubled when the number of RAW cells was doubled. Comparing (c) and (d), the production of TNFα was reduced even with the later addition of macrophages.

****P<0.0001(one-way ANOVA followed by Tukey's post hoc test). The data is presented as the mean ± SEM. GTS-21, 3-(2,4-Dimethoxybenzylidene)- anabaseine dihydrochloride; LPS, lipopolysaccharide; TNF-α, tumor necrosis factor α; ANOVA, analysis of variance; SEM, standard error of the mean.

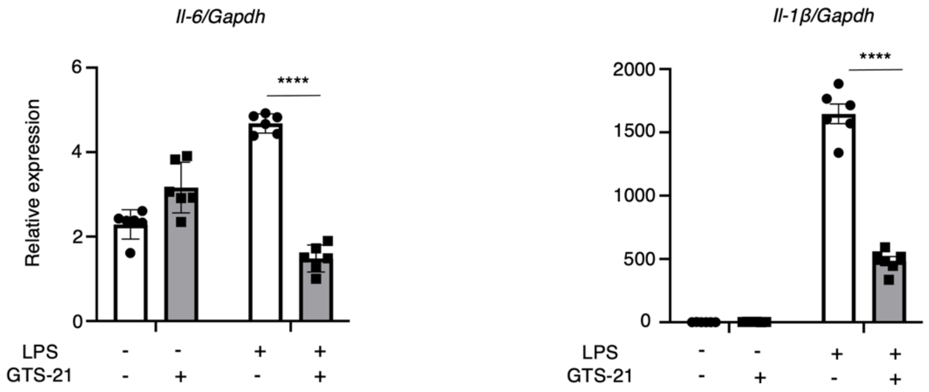

Supplementary Figure 8. GTS-21 decreased inflammatory cytokines.

RAW 264 cells were treated with GTS-21 (100 μM) for 15 min prior to LPS (100 ng ml^{-1}) stimulation for 4h, then cells were collected and qPCR was conducted.

Both Il-6 and Il-1β expression levels elevated by LPS administration were decreased by GTS-21.

****P<0.0001(Two-way ANOVA followed by Tukey's post hoc test). The data is presented as the mean ± SEM. GTS-21, 3-(2,4-Dimethoxybenzylidene)- anabaseine dihydrochloride; LPS, lipopolysaccharide; qPCR, quantitative polymerase chain reaction; ANOVA, analysis of variance; SEM, standard error of the mean.

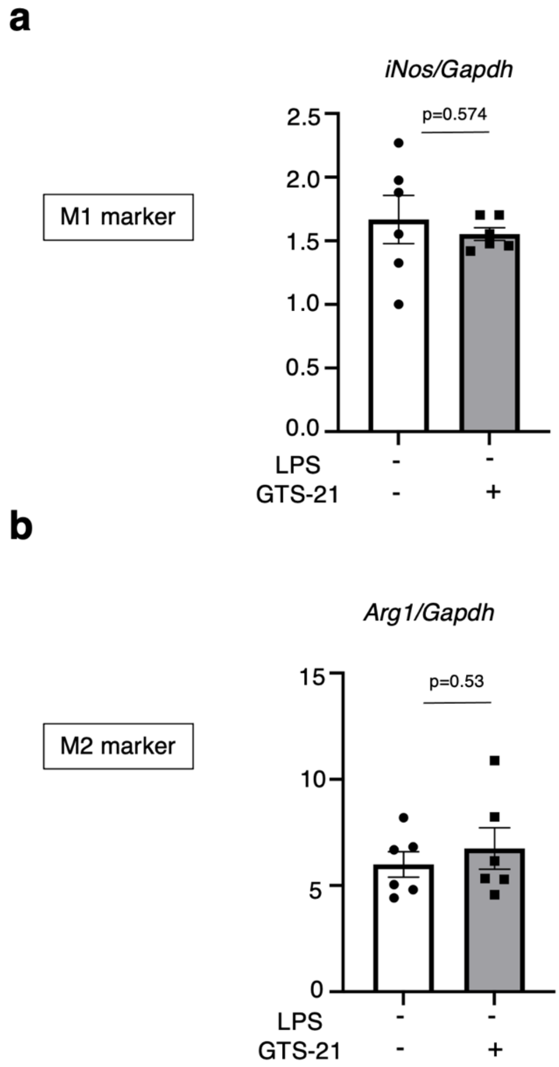

Supplementary Figure 9. Phenotypic changes of macrophages induced by GTS-21.

RAW 264 cells were treated with GTS-21 (100 μM) for 15 min prior to LPS (100 ng ml^{-1}) stimulation for 4h, then cells were collected and qPCR was conducted.

GTS-21 showed a decreasing trend in iNOS (M1 marker, inflammatory) and an increasing trend in Arg1 (M2 marker, anti-inflammatory), but no significant difference was observed (P=0.574 and P=0.53, iNos and Arg1, respectively; Unpaired T-test). GTS-21, 3-(2,4-Dimethoxybenzylidene)- anabaseine dihydrochloride; LPS, lipopolysaccharide; qPCR, quantitative polymerase chain reaction.

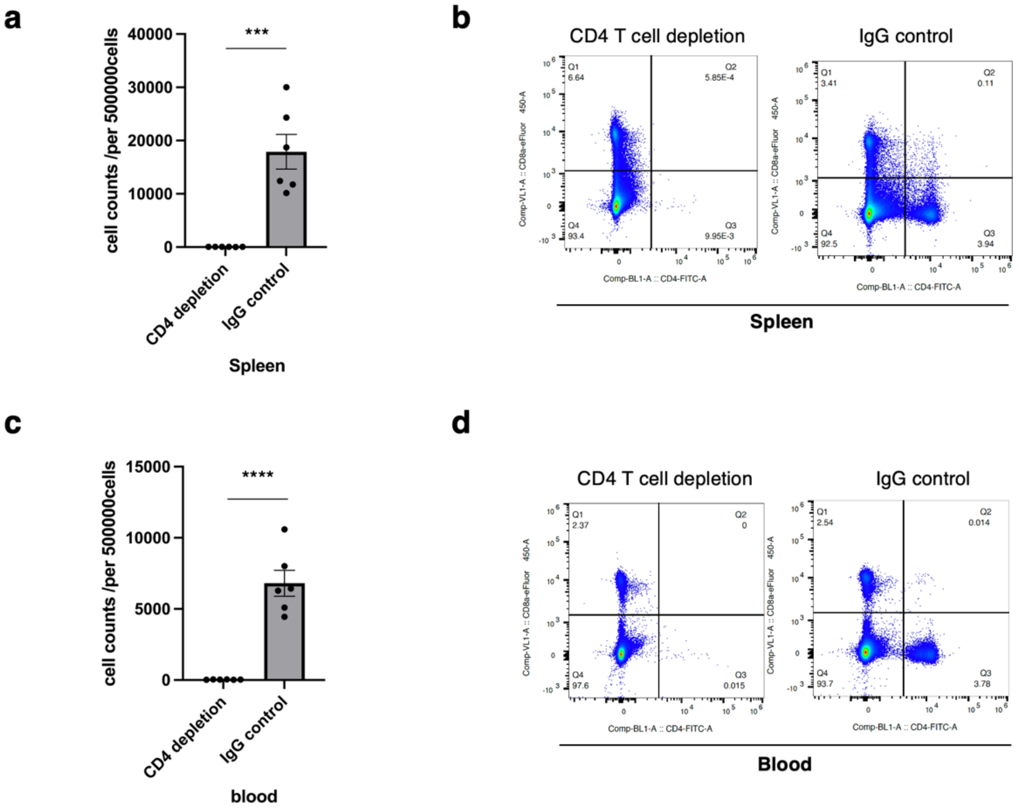

Supplementary Figure 10. Efficacy of CD4+ T cells depletion.

The efficiency of CD4^{+} T cells depletion was confirmed by Flow cytometry of spleen and whole blood samples removed from each treated mouse.

(a) Number of CD4^{+} T cells in the spleen. (38.67±8.14 and 17900±3429 cells per 50000 cells for CD4+ T cell depletion with anti-CD4 antibody administration and IgG control administration, respectively; n = 6; P = 0.0003)

(c) Number of CD4^{+} T cells in the whole blood. (31.00±9.21 and 6897±907.5 cells per 50000 cells for CD4+ T cell depletion and IgG control, respectively; n = 6; P<0.0001).

Representative image of the results of Flow cytometry of spleen (b) and whole blood (d) sample.

***P<0.0001, ****P<0.0001. The data are expressed as the mean ± SEM and analyzed by unpaired T-test. SEM, standard error of the mean.

Supplementary Tables

Supplementary Table 1. The primer sequencing used in tail PCR

Primer type	Sequence 5'→3'
Mutant	CCCAGAAATGCCAGATTACG
Common	CTTGGGCTGCCAGAATTTCTC
Wild-type	TTACAGTCGGCCAGGCTGAC
Chrna7_exon4 Fw	TCTGGCCATCTGGAAAACGA
Chrna7_exon4 Rv	ACATGTCTGAGTACCCCGGA

Supplementary Table 2. The primer sequencing used in RT-PCR experiments

Gene name	Forward (5'→3')	Reverse (5'→3')
mGapdh	AGGTCGGTGTGAACGGATTTG	TGTAGACCATGTAGTTGAGGTCA
mNgal	TCTCTGTCCCCACCGACCAA	ACAGGTGGATGGGGAGTGCT
mKim-1	ACATATCGTGGAATCACAACGAC	ACTGCTCTTCTGATAGGTGACA
mIl-6	AGGATGCACATCAAAAGGCTT	GGCCTCGGTTAGGAAGGATAC
mIl1-β	AGCAGCATCACCTTCGCTTAG	GTGTCCAGATATTGGCATGGG
mNos2(iNos)	CACCTTGGAGTTCACCCAGT	ACCACTCGTACTTGGGATGC
mArg1	CATGGGCAACCTGTGTCCTT	TCCTGGTACATCTGGGAACTTTC
