# Supplementary material for: Alpha 7 nicotinic acetylcholine receptors signaling boosts cell-cell interactions in macrophages effecting anti-inflammatory and organ protection
Source: Commun Biol. 2023 Jun 23;6:666. doi: 10.1038/s42003-023-05051-2 (PMC10290099; doi:10.1038/s42003-023-05051-2)
Supplement: Supplementary file 5 — Reporting Summary [file 42003_2023_5051_MOESM5_ESM.pdf]

nature portfolio

Corresponding author(s): Tsuyoshi Inoue

Last updated by author(s): June, 14, 2023

Reporting Summary

Nature Portfolio wishes to improve the reproducibility of the work that we publish. This form provides structure for consistency and transparency in reporting. For further information on Nature Portfolio policies, see our Editorial Policies and the Editorial Policy Checklist.

Statistics

For all statistical analyses, confirm that the following items are present in the figure legend, table legend, main text, or Methods section.

n/a	Confirmed	
☐	☒	The exact sample size (n) for each experimental group/condition, given as a discrete number and unit of measurement
☐	☒	A statement on whether measurements were taken from distinct samples or whether the same sample was measured repeatedly
☐	☒	The statistical test(s) used AND whether they are one- or two-sided<br>*Only common tests should be described solely by name; describe more complex techniques in the Methods section.*
☐	☒	A description of all covariates tested
☐	☒	A description of any assumptions or corrections, such as tests of normality and adjustment for multiple comparisons
☐	☒	A full description of the statistical parameters including central tendency (e.g. means) or other basic estimates (e.g. regression coefficient) AND variation (e.g. standard deviation) or associated estimates of uncertainty (e.g. confidence intervals)
☐	☒	For null hypothesis testing, the test statistic (e.g. F, t, r) with confidence intervals, effect sizes, degrees of freedom and P value noted<br>*Give P values as exact values whenever suitable.*
☒	☐	For Bayesian analysis, information on the choice of priors and Markov chain Monte Carlo settings
☒	☐	For hierarchical and complex designs, identification of the appropriate level for tests and full reporting of outcomes
☒	☐	Estimates of effect sizes (e.g. Cohen's d, Pearson's r), indicating how they were calculated

Our web collection on statistics for biologists contains articles on many of the points above.

Software and code

Policy information about availability of computer code

Data collection	Quantitative Real-Time PCR ; CFX Maestro Software 1.1<br>Flow Cytometry ; Attune Software 5.1.1, Single-cell RNA sequencing ; Illumina Novaseq platform
Data analysis	Flow Cytometry ; Flow Jo 10.8.2, Plots ; Graph Pad Prism 9<br>Single-cell RNA sequencing ; Seurat v.4.0.6, Single R v.1.4.1, CellPhoneDB, NicheNet R package

For manuscripts utilizing custom algorithms or software that are central to the research but not yet described in published literature, software must be made available to editors and reviewers. We strongly encourage code deposition in a community repository (e.g. GitHub). See the Nature Portfolio guidelines for submitting code & software for further information.

Data

Policy information about availability of data

All manuscripts must include a data availability statement. This statement should provide the following information, where applicable:

- Accession codes, unique identifiers, or web links for publicly available datasets
- A description of any restrictions on data availability
- For clinical datasets or third party data, please ensure that the statement adheres to our policy

Raw sequencing data were deposited in the DDBJ Sequence Read Archive under the accession numbers DRA014634 and DRA016213. The sequenced data were deposited in the Gene Expression Omnibus (GEA) under accession number E-GEAD-529, E-GEAD-608 and E-GEAD-609.

March 2021

Human research participants

Policy information about studies involving human research participants and Sex and Gender in Research.

Reporting on sex and gender	N/A
Population characteristics	N/A
Recruitment	N/A
Ethics oversight	N/A

Note that full information on the approval of the study protocol must also be provided in the manuscript.

Field-specific reporting

Please select the one below that is the best fit for your research. If you are not sure, read the appropriate sections before making your selection.

☒ Life sciences ☐ Behavioural & social sciences ☐ Ecological, evolutionary & environmental sciences

For a reference copy of the document with all sections, see nature.com/documents/nr-reporting-summary-flat.pdf

Life sciences study design

All studies must disclose on these points even when the disclosure is negative.

Sample size	For Wild-type mice experiments, we used male mice based on previous reports using LPS sepsis model (PMID:29118926). In LysMCre:α7flox mice, we increased the number of mice around 10 or more based on Guyot et al. (PMID:30885844) because of high variability.
Data exclusions	No data exclusions in the study.
Replication	All experiments excluded single-cell RNA sequencing were repeated at least three times independently. The samples used for single-cell RNA sequencing were those that showed reproducibility of at least three times under the same experimental conditions and were submitted for sequencing.
Randomization	Mice were randomly divided assigned to the experimental assistant and numbered for use in the study.
Blinding	LysMCre:α7flox mice were randomly assigned into the groups and labeled with numbers by experimental assistant. The experimenters administrated the drugs without which mice is knockout or littermate wild-type.

Behavioural & social sciences study design

All studies must disclose on these points even when the disclosure is negative.

Study description	N/A
Research sample	N/A
Sampling strategy	N/A

Data collection	N/A
Timing	N/A
Data exclusions	N/A
Non-participation	N/A
Randomization	N/A

Ecological, evolutionary & environmental sciences study design

All studies must disclose on these points even when the disclosure is negative.

Study description	N/A
Research sample	N/A
Sampling strategy	N/A
Data collection	N/A
Timing and spatial scale	N/A
Data exclusions	N/A
Reproducibility	N/A
Randomization	N/A
Blinding	N/A

Did the study involve field work? ☐ Yes ☐ No

Field work, collection and transport

Field conditions	N/A
Location	N/A
Access & import/export	N/A
Disturbance	N/A

Reporting for specific materials, systems and methods

We require information from authors about some types of materials, experimental systems and methods used in many studies. Here, indicate whether each material, system or method listed is relevant to your study. If you are not sure if a list item applies to your research, read the appropriate section before selecting a response.

Materials & experimental systems

n/a	Involved in the study
☐	☒ Antibodies
☐	☒ Eukaryotic cell lines
☒	☐ Palaeontology and archaeology
☐	☒ Animals and other organisms
☒	☐ Clinical data
☒	☐ Dual use research of concern

Methods

n/a	Involved in the study
☒	☐ ChIP-seq
☐	☒ Flow cytometry
☒	☐ MRI-based neuroimaging

Antibodies

Antibodies used

For Flow cytometry (refer to supplementary methods): Anti-mouse CD16/32 (2.4G2),BD. Anti-mouse CD4-FITC (GK1.5),Themo Fisher. Anti-mouse CD8a-eFloro 450 (53-6.7), Thermo Fisher. 7AAD, Thermo Fisher.

Validation

All antibodies used in this study have been validated by manufacturer and have been used according to manufacture's instructions.

Eukaryotic cell lines

Policy information about cell lines and Sex and Gender in Research

Cell line source(s)

Refer to Methods section "Cell culture". We used newly obtained RAW 264 (RIKEN BRC CELL bank, Ibaraki, Japan) and U937 (JCRB Cell Bank, Osaka, Japan) in this experiments.

Authentication

RAW 264 were obtained from RIKEN BRC CELL bank (https://cell.brc.riken.jp/en/), and U937, were obtained from JCRB Cell Bank (https://cellbank.nibiohn.go.jp/english/), that provides authentication documents.

Mycoplasma contamination

Only newly obtained cell lines negative for mycoplasma were used.

Commonly misidentified lines
(See ICLAC register)

No misidentified lines were used in this study.

Palaeontology and Archaeology

Specimen provenance

N/A

Specimen deposition

N/A

Dating methods

N/A

☐ Tick this box to confirm that the raw and calibrated dates are available in the paper or in Supplementary Information.

Ethics oversight

N/A

Note that full information on the approval of the study protocol must also be provided in the manuscript.

Animals and other research organisms

Policy information about studies involving animals; ARRIVE guidelines recommended for reporting animal research, and Sex and Gender in Research

Laboratory animals

Refer to Method section "Animal experiments". Animal experiments were conducted in accordance with the Guidelines for the Care and Use of Laboratory Animals, approved by the University of Tokyo Graduate School of Medicine (M-P18-051) and Nagasaki University (2006101636). C57BL/6J male mice (8 -12 weeks old, purchased from CLEA) were used as wild-type mice for LPS/ GTS-21 experiments. LysMCre:α7flox mice were generated by crossbred lysozyme M (LysM)-Cre and α7AChR flox/flox (Jackson Laboratory). They were used in LPS/ GTS-21 experiments (male and female, 8 -18 weels old).

Wild animals	N/A
Reporting on sex	We exclusively employed male mice for wild-type experiments, as previous studies (PMID:29118926) utilizing LPS injection model had also used male mice. In experiments using LysMCre:α7flox mice, both male and female mice used to increase the number of experiments, as this mice strain was known to have high variability in Guyot et al. studies (PMID:30885844).
Field-collected samples	N/A
Ethics oversight	Animal experiments were conducted in accordance with the Guidelines for the Care and Use of Laboratory Animals, approved by the University of Tokyo Graduate School of Medicine (M-P18-051) and Nagasaki University (2006101636).

Note that full information on t

Clinical data

Policy information about clinical studies

All manuscripts should comply with the ICMJE guidelines for publication of clinical research and a completed CONSORT checklist must be included with all submissions.

Clinical trial registration	N/A
Study protocol	N/A
Data collection	N/A
Outcomes	N/A

Dual use research of concern

Policy information about dual use research of concern

Hazards

Could the accidental, deliberate or reckless misuse of agents or technologies generated in the work, or the application of information presented in the manuscript, pose a threat to:

No	Yes	
☒	☐	Public health
☒	☐	National security
☒	☐	Crops and/or livestock
☒	☐	Ecosystems
☒	☐	Any other significant area

Experiments of concern

Does the work involve any of these experiments of concern:

No	Yes	
☒	☐	Demonstrate how to render a vaccine ineffective
☒	☐	Confer resistance to therapeutically useful antibiotics or antiviral agents
☒	☐	Enhance the virulence of a pathogen or render a nonpathogen virulent
☒	☐	Increase transmissibility of a pathogen
☒	☐	Alter the host range of a pathogen
☒	☐	Enable evasion of diagnostic/detection modalities
☒	☐	Enable the weaponization of a biological agent or toxin
☒	☐	Any other potentially harmful combination of experiments and agents

ChIP-seq

Data deposition

☐ Confirm that both raw and final processed data have been deposited in a public database such as GEO.

☐ Confirm that you have deposited or provided access to graph files (e.g. BED files) for the called peaks.

Data access links<br>*May remain private before publication.*	N/A
Files in database submission	N/A
Genome browser session<br>(e.g. UCSC)	N/A

Methodology

Replicates	N/A
Sequencing depth	N/A
Antibodies	N/A
Peak calling parameters	N/A
Data quality	N/A
Software	N/A

Flow Cytometry

Plots

Confirm that:

☒ The axis labels state the marker and fluorochrome used (e.g. CD4-FITC).

☒ The axis scales are clearly visible. Include numbers along axes only for bottom left plot of group (a 'group' is an analysis of identical markers).

☒ All plots are contour plots with outliers or pseudocolor plots.

☒ A numerical value for number of cells or percentage (with statistics) is provided.

Methodology

Sample preparation	Refer to Supplementary Methods section " Validation of CD4 positive T cell depletion".
Instrument	Attune NxT (Thermo Fisher Scientific)
Software	Flow Jo 10.8.2
Cell population abundance	We analyzed both spleen and whole blood samples rich in lymphocytes with dead cells removed using 7AAD antibody.
Gating strategy	From the lymphocyte rich cluster, singlets were gated based on forward scatter and side scatter. Live cells were selected using 7AAD. And finally CD4 positive- CD8 negative cells were defined as CD4 positive cells.

☐ Tick this box to confirm that a figure exemplifying the gating strategy is provided in the Supplementary Information.

Magnetic resonance imaging

Experimental design

Design type	N/A

Design specifications	N/A
Behavioral performance measures	N/A

Acquisition

Imaging type(s)	N/A
Field strength	N/A
Sequence & imaging parameters	N/A
Area of acquisition	N/A

Diffusion MRI ☐ Used ☐ Not used

Preprocessing

Preprocessing software	N/A
Normalization	N/A
Normalization template	N/A
Noise and artifact removal	N/A
Volume censoring	N/A

Statistical modeling & inference

Model type and settings	N/A
Effect(s) tested	N/A

Specify type of analysis: ☐ Whole brain ☐ ROI-based ☐ Both

Statistic type for inference (See Eklund et al. 2016)	N/A
Correction	N/A

Models & analysis

n/a	Involved in the study
☐	☐ Functional and/or effective connectivity
☐	☐ Graph analysis
☐	☐ Multivariate modeling or predictive analysis

Functional and/or effective connectivity	N/A
Graph analysis	N/A
Multivariate modeling and predictive analysis	N/A
